# Pro-Diversity Beliefs and the Diverse Person’s Burden

**DOI:** 10.1007/s11229-022-03785-w

**Published:** 2022-08-22

**Authors:** Daniel Steel, Karoline Paier

**Affiliations:** 1 W. Maurice Young Centre for Applied Ethics, University of British Columbia , Vancouver, Canada; 2 Department of Philosophy, University of British Columbia, Vancouver, Canada

**Keywords:** Diversity, Ignorance, Positive stereotypes, Epistemic injustice, Ideology

## Abstract

Pro-diversity beliefs hold that greater diversity leads to better results in academia, business, politics and a variety of other contexts. This paper explores the possibility that pro-diversity beliefs can generate unfair expectations that marginalized people produce distinctive bonuses, a phenomenon we refer to as the “diverse person’s burden”. We suggest that a normic conception of diversity, according to which non-diversity entails social privilege, together with empirical research on psychological entitlement suggests an explanation of how the diverse person’s burden can arise in many social settings. We also suggest structural and institutional remedies to address the diverse person’s burden, as well as an individual virtue we label positional awareness.

## Introduction

Pro-diversity beliefs, according to which diversity is beneficial and likely to produce better results in academia, business, politics, and elsewhere, might seem entirely beneficial and even morally laudatory. After all, positive attitudes toward inclusiveness and diversity are obviously better than overt discrimination and exclusion of out-groups. Furthermore, some empirical research has found that diverse groups tend to perform better when their members hold pro-diversity beliefs (Homan et al., 2007; van Knippenberg et al., 2013), so such beliefs may be beneficial from an epistemic as well as ethical perspective. However, in this paper we explore the possibility that pro-diversity beliefs can also have ethical and epistemic downsides. Specifically, we develop a suggestion by Phillips (2017) that pro-diversity beliefs can lead to unfair expectations that members of minority or discriminated against groups produce distinctive bonuses not expected of their non-diverse counterparts.

Phillips expresses this idea in connection with the “business case for diversity,” which claims that diversity should be promoted because more diverse groups produce superior results.

The logic of the business case for diversity rests on the idea that those who deviate from the norm must demonstrate that their presence in the organization will result in the company making more money. Imagine walking into a company and having the burden resting on your shoulders to prove that you can make the company a million more dollars in profit. That is a heavy burden to bear. We should reflect on how seeking evidence of a business case for diversity reifies the status quo and legitimates the idea that some people belong and deserve to be included in organizations, while other people have to go above and beyond to prove their worth. The questions we ask about diversity have power. They have underlying assumptions that often go unspoken. (Phillips, 2017, p. 244)

While Phillips’ claims here are made within a business context, the idea easily transposes to other walks of life. For example, in academia the heightened expectations might be couched in terms of research output. And in any context, the extra expectations may concern work related to the individual’s perceived status as a “diverse person,” such as taking the lead on initiatives to increase workplace inclusion and diversity or being expected to speak on behalf of one’s demographic group. For convenience, we use the term “diverse person’s burden” to refer to heightened expectations that may be experienced by marginalized individuals and the stresses these expectations engender. We interpret the passage from Phillips quoted above as suggesting that pro-diversity beliefs can, perhaps unintentionally, lead to the diverse person’s burden.

Several philosophers have discussed issues related to pro-diversity beliefs and the diverse person’s burden. In *The Imperative of Integration*, Anderson claims that what she calls the “diversity model” can, when detached from concerns about social justice, promote racial stereotyping (2013, pp.142–143). Fehr (2011) and Berenstain (2016) discuss uncompensated diversity work that marginalized people are often expected to perform, while Davis (2016) argues that prejudicial credibility excesses—in which marginalized individuals are assumed to have knowledge about certain subjects related to their social identities—are a type of epistemic injustice. Our work advances this literature by examining the diverse person’s burden as a general phenomenon in its own right, and suggesting an underlying mechanism that links a variety of different cases from different contexts (e.g., academia, business, etc.).

Our approach to the diverse person’s burden rests on a distinction between two concepts of diversity. The first understands diversity as variety within a group while the second emphasizes the inclusion of people who are subject to discrimination and social marginalization. The first diversity concept is labeled “mere diversity” by Harding (2015, p. 35) and “egalitarian diversity” by Steel et al., (2018). Egalitarian diversity concepts are commonly used in empirical work on diversity (Steel et al., 2018). They are also often associated with the idea that demographic differences (e.g., in ethnicity, race, gender, religion, socio-economic status, etc.) may correlate with distinct perspectives, knowledge, or experiences that combine to promote superior results (Anderson, 2006; Longino, 1990; Page, 2007, 2017).

The second understanding of diversity we discuss is labeled “normic diversity” by Steel et al., (2018). Normic diversity assumes a non-diverse norm, or “mythical norm” to use Lorde’s expression (Lorde, 1984, p. 111), such as heterosexual able-bodied cisgender white male, and defines diversity as deviation from that norm (cf. Medina, 2013, p. 51). Importantly for our purposes, the non-diverse norm picks out a segment of the population that enjoys privileged social status. A diverse person, by contrast, is someone who belongs to one or more groups that have been persistently marginalized or discriminated against. According to the normic conception, then, a group is diverse to the extent that diverse persons are included among its members (cf. Harding, 2015, p. xi). Normic diversity is often linked to explanations that focus on distorting epistemic and moral psychological effects of social privilege (Harding, 2004, 2015; Intemann, 2010; Rolin, 2016; Wylie, 2011).

Non-diversity in a normic sense entails social privilege. In this paper, we claim that privilege is crucial to generating the diverse person’s burden because of its tendency to promote psychological entitlement, a persistent sense of oneself as especially deserving (Campbell et al., 2004). We suggest that enhanced psychological entitlement among privileged individuals motivates beliefs that inequalities that benefit them are fair, which in turn generate expectations that diverse persons produce distinctive bonuses to justify inclusion. Which bonuses are expected can take many different forms depending on the context, so this is a general mechanism whereby pro-diversity beliefs can be harmful. We propose structural, institutional, and individual approaches for addressing the diverse person’s burden.

The structure of the paper will be as follows. In Sect. 2, we discuss philosophical literature that has addressed some aspects of the diverse person’s burden (Anderson, 2013; Berenstain, 2016; Davis, 2016; Fehr, 2011), as well as work describing the experiences of those involved in diversity training (Ahmed 2008; DiAngelo 2018). Section 3 presents our analysis of the diverse person’s burden, framing it in connection with literature on psychological entitlement (Campbell et al., 2004té et al. 2021; Grubb & Exline, 2016; Zitek & Schlund, 2020). In Sect. 4, we consider what should be done about the diverse person’s burden in light of our analysis. Section 5 concludes.

## The Diverse Person’s Burden

While the phrase “diverse person’s burden” has not, to our knowledge, been previously used, ideas relating to it have been discussed. In this section, we examine philosophical literature that discusses ways in which pro-diversity beliefs can create unfair expectations for members of marginalized groups. Then we turn to related works that discuss experiences of those involved in diversity and inclusion initiatives or training.

Anderson’s (2013) discussion of the “diversity model” is a good place to start. The diversity model is essentially the business case for diversity used to argue for affirmative action, that is, to advocate for policies that aim to increase numbers of marginalized individuals in hiring, university admission, and the awarding of government contracts (Anderson, 2013, Chap. 7). Thus, the diversity model argues that, by increasing racial diversity, affirmative action stands to promote epistemic diversity and thereby yield superior outcomes in the workplace and education. While Anderson recognizes some positive aspects of the diversity model (Anderson, 2013, p. 141), she is ultimately critical of it:

...the diversity model, when separated from justice concerns, promotes racial myths that may be stigmatizing. In stressing how different African Americans are from other Americans, it potentially primes stereotypes of blacks as alien and blocks recognition of common perspectives and identities. (Anderson 2013, p. 142–143)

Anderson’s claim that the diversity model can promote stigmatizing racial stereotypes is related to the diverse person’s burden insofar as it identifies a potentially negative impact of a pro-diversity belief. However, unlike Phillips’ statement, Anderson does not discuss potentially harmful impacts of heightened expectations for marginalized individuals. Yet the belief that racial diversity yields epistemic advantages is often associated with the idea that racialized individuals tend to possess valuable distinctive perspectives stereotypically associated with their group. The next author we consider takes up this idea.

Davis (2016) argues that positive stereotypes (i.e., flawed generalizations about social minorities which are perceived to be innocent or even complementary by the dominant group) can lead to epistemic injustices. Examples of positive stereotypes include African-Americans as particularly athletic, women as caring, gay men as fashionable or disabled people as inspiring. A literature review by Czopp and colleagues suggests that positive stereotypes can be psychologically harmful to their targets, impair intergroup relationships, and reinforce social inequalities (Czopp et al., 2015, pp. 451, 457). ​​While Fricker (2007) explains how negative stereotypes about race and gender and the resulting credibility deficits generate epistemic injustices, Davis (2016) makes the case that positive stereotypes can result in credibility excesses, which can also generate specific epistemic harms.

Consider, for instance, one woman in a room full of men being asked to explain feminism, regardless of whether she is in fact a feminist or has knowledge about feminism. Here, the epistemic agent receives a credibility excess due to her presumed social category and the related positive stereotype. Davis suggests that, in addition to psychological harms, such as anxiety about living up to unwanted expectations, these types of situations produce epistemic harms for the diverse person, namely “the harm from compulsory representation” (Davis, 2016, p. 6). In cases of compulsory representation, the diverse person’s epistemic contributions are acceptable to the non-diverse audience only if they conform to positive stereotypes. As Davis puts it, “The problem […] is not that one is not permitted to contribute in ways that are perceived to extend beyond dominant experiences; rather, the problem is that one is only permitted (and expected to) contribute in ways that are considered ‘unique’ and ‘distinct’” (Davis, 2016, p. 6). Davis suggests that this involves a type of tokenism in which an agent is invited to participate in an epistemic exchange only as a representative of a demographic category and not as an individual. The underlying mechanism of these harms has been described as epistemic othering (Davis, 2016, p. 5; Pohlhaus 2014) or epistemic objectification (McGlynn, 2020), both of which emphasize that the diverse person is treated as interchangeable with others of their demographic group and therefore harmed in their epistemic agency.

Moreover, compulsory representation can be a form of epistemic exploitation (Berenstain, 2016; Davis, 2016; Fehr, 2011). Fehr argues that whenever a person is used as “a source of diversity,” this constitutes “epistemic diversity work” (2011, p. 141). Furthermore, diverse people can be considered exploited when they are repeatedly allocated uncompensated epistemic labor related to their social category. For example, this can happen when marginalized people are expected to educate others in their organization on the nature of their oppression (Berenstain, 2016, p. 570). Berenstain calls this a “burden on the marginalized to educate and enlighten” (p. 571). Berenstain explains that there are three features which render these interactions exploitative: “opportunity costs associated with the labor of educating the oppressor, the double bind that marginalized people find themselves in when faced with the demand to educate, and the default skeptical responses from the privileged when the marginalized do acquiesce and fulfill their demands” (Berenstain, 2016, p. 572). Consider these three features in turn.

The opportunity costs are easy to appreciate: the person could be doing something else with their time that may be more enjoyable, rewarding, or productive in terms of advancing their career. And even if this educational labor may inspire some members of the non-diverse group, it still remains an extra burden for the diverse person: “the marginalized have still sacrificed their time, energy, and expertise in the service of the privileged” (Berenstain, 2016, p. 575). Second, invitations to provide such education can come with a double bind: often diverse people cannot simply disengage from these interactions and may risk being labeled “difficult” or “rude” if they decline to explain themselves. They may encounter irritation and frustration from the non-diverse hearer, and may risk affirming a negative stereotype. Fehr (2011) recognizes this double bind too. Fehr writes that rejecting and accepting diversity work can be risky: “Refusal is risky because it involves withholding services from a person with greater power and authority than the diversity worker or from a community in which one is a marginal member” (2011, pp. 142). Moreover, accepting diversity work can also be risky: “Epistemic diversity work can involve telling people things that they might not be inclined to hear. The power differences among members of formal and informal epistemic communities have professional and epistemic consequences” (Fehr, 2011, p. 143).

Third, when the diverse person offers testimony of their experience, it often will be met with default skepticism by the non-diverse hearer. Berenstain argues that these skeptical responses falsely position the dominant hearer as an epistemic peer concerning oppressive experiences. This creates an additional demand to respond for the marginalized person as well as the risk of being seen as “losing the debate” if one does not answer “correctly” (Berenstain, 2016, pp.579–580). Such circumstances can lead to “gaslighting,” wherein repeated skepticism from members of privileged groups leads marginalized individuals to doubt their own experiences, and to hesitancy about sharing those experiences in the first place (Berenstain, 2016, pp. 580–581; cf. Dotson, 2011).

Another aspect of the diverse person’s burden can be found in Dotson’s (2012b) paper “How is this paper philosophy?”. As the title suggests, marginalized individuals are more likely to be asked to provide extra justification as to how their respective endeavors fit within a field or organization. This paper specifically targets exclusionary mechanisms in philosophy, yet it is easy to imagine similar dynamics in other contexts. Dotson (2012b) discusses how historical power structures affect what is seen as a legitimate project and how much justification is needed. There are two insights which are particularly noteworthy for our discussion: first, this extra demand for justification rests on field specific norms which are skewed in favor of the dominant group. This means that marginalized groups have to perform an extra effort to justify their projects in line with the norms of the historically powerful.

Furthermore, according to Dotson, marginalized groups are more likely to rightfully question and deny these norms. Consequently, this can make it difficult for the diverse person to justify their interests in line with traditional norms and, yet again, puts the burden of changing these norms on them.

All of these harmful social and epistemic practices are furthermore closely tied to certain types of ignorance which arise due to the social position of an epistemic agent (e.g. Dotson 2012a; Pohlhaus 2012). There are two types of ignorance which are particularly relevant in this context: (1) ignorance in terms of neglecting and dismissing certain epistemic resources and (2) ignorance which actively distorts and misrepresents parts of the social reality. To illustrate these two types of ignorance, consider Dotson’s point that marginalized groups have always developed so-called “alternative epistemologies, countermythologies, and hidden transcripts” (Dotson, 2012a, p. 31). Failure by members of non-marginalized groups to acknowledge and consider these epistemic resources constitutes willful hermeneutical ignorance (Pohlhaus 2012), which can result in distorted perceptions of social reality and contributory epistemic injustice (Dotson, 2012a).

The second type of ignorance actively misunderstands and misrepresents the world. This phenomenon has been described by Medina (2013) and Mills (2007). For example, Mills coins the term “white normativity ” to refer to “a white refusal to recognize the long history of structural discrimination that has left whites with the differential resources they have today, and all of its consequent advantages in negotiating opportunity structures” (2007, p. 28). As Mills (2007) points out, active ignorance of this kind is often expressed in the ableist vernacular of “color blindness.” Active ignorance typically involves the creation of narratives purporting to justify inequalities and comes with a variety of defense strategies and mechanisms (Medina, 2013) that cannot be undone by reasoned arguments alone. According to Medina, it is “an ignorance that is not easy to undo and correct, for this requires––the reconfiguration of epistemic attitudes and habits––as well as social change” (Medina, 2013, p. 39). Active ignorance of the sort described by Mills and Medina is closely related to Berenstain’s observations about the negative responses that marginalized individuals often face when raising issues about oppression.

The social dynamics described above are attested to in work describing the experiences of people for whom promoting diversity and inclusion within organizations is a profession (Ahmed, 2012; DiAngelo, 2018). In her (2012) book, *On Being Included*, Ahmed describes her own experience of being encouraged, as a female, racialized scholar, to undertake diversity and inclusion initiatives as well as her subsequent research on the experiences of diversity officers in institutions of higher education in England and Australia. Ahmed’s discussion illustrates several of the points raised above. Similar to Anderson’s criticisms of the diversity model, Ahmed critiques diversity rhetoric as a way of papering over social injustices and promoting a “food court” perspective in which diverse people deliver exotic spices for the enjoyment of a privileged majority (2012, pp. 66+; cf. hooks 1992, pp. 21–39). In addition, Ahmed’s discussion echoes many of the themes raised by Fehr, Davis, and Berenstain. Ahmed notes that diversity officers at institutions of higher education tend to be female and/or racialized minorities, and recalls her own experience of being recruited to join a “race equality team” in which the only other faculty member was also a person of color (2012, p. 4). She notes that creation of diversity offices or other similar initiatives in response to legal or administrative requirements are often not accompanied by interest within the institution in taking substantive action (2012, p. 23). Moreover, Ahmed reports that experience of resistance to diversity promotion, often described as “a brick wall” or “institutional inertia,” was common among her sample of interviewees (2012, pp. 26–27). Ahmed develops the concept of “non-performative” speech acts to characterize officially expressed commitments to diversity that do not involve any specific action and which may even help to conceal ongoing discrimination and other inequalities (2012, pp. 116–117). For example, she describes how official diversity commitments can lead to push back when concerns about racism are raised: since the institution is committed to diversity, concerns about racism within its ranks are a public relations problem (2012, pp. 143–147). Consequently, those who draw attention to racism risk becoming the target of a counteroffensive that seeks to restore the positive image. As Ahmed puts it, “one of the best ways you can deflect attention from racism is to hear racism as an accusation” (2012, p. 150).

The book, *White Fragility*, by DiAngelo (2018), explores this last idea in detail. The term “white fragility” refers to defensive reactions often exhibited by white people when topics of race or racism are broached, reactions which DiAngelo frequently encountered in her work as a diversity consultant. Examples include assertions of non-racism (e.g., “I don’t see color,” “I was raised to treat everyone the same,” etc.) or personal details (e.g., “I grew up poor”; “I have black family members”; “I marched in the 60’s,” etc.) presented as evidence that one is somehow exempt from the forces of race and racism in society. White fragility is also often expressed by counterclaims that whoever raised the issue of racism is the real racist. DiAngelo links white fragility to an understanding of racism as limited to derogatory beliefs about, or malicious actions towards, people of other racialized groups. Given this understanding, concerns about racism are likely to be interpreted as personal accusations, a reaction that forecloses discussion of how historical and current systemic racism shapes society and the experiences of everyone in it. According to DiAngelo, suppressing frank discussions of race and thereby helping to maintain the unequal racial status quo is the social function of white fragility.

DiAngelo’s discussion of white fragility, therefore, echoes points made above about ignorance and stresses faced by marginalized individuals who attempt to educate members of a majority group about their experiences of social inequality. Consider a concrete example of this phenomenon described in a report on anti-Indigenous discrimination within the health care system of the Canadian province British Columbia:

What we heard time and time again from Indigenous medical students is that they are not only having to educate their peers about the colonizing history of Canada – past and present – but they are also having to educate their preceptors and their teachers. What’s more is that not only are our preceptors ignorant of this history, they often directly disagree with it. This subjects us to quite traumatizing situations that force us to take time off of school. (Turpel-Lafond et al. 2020, p. 96)

This strikes us as a clear example of epistemic exploitation and the diverse person’s burden.

The literature discussed in this section illustrates several ways in which pro-diversity beliefs can promote expectations that marginalized individuals produce distinctive bonuses stereotypically related to their perceived identities, which can in turn have a variety of harmful consequences, including negative reactions from members of the majority group. However, none identify or analyze the diverse person’s burden––the heightened expectations marginalized people face––as a general phenomenon in its own right, and most focus on situations in which diverse people are expected to provide education about oppression or discrimination. As a result, prior work has not examined the potential similarity between Phillip’s concern with expectations of financial diversity bonuses in business contexts and worries about prejudicial credibility excesses as a form of epistemic injustice. In what follows, we propose a more general account of the diverse person’s burden that is applicable in a variety of contexts. In Sect. 3, we show how examining the diverse person’s burden as a general phenomenon leads to an understanding of dynamics shared by the variety of examples discussed above. In addition to its inherent intellectual interest, this understanding suggests structural, institutional, and individual level measures for counteracting the diverse person’s burden that we describe below in Sect. 4.

## A diagnosis of the Diverse Person’s Burden

Our analysis of the diverse person’s burden is founded on a distinction between *egalitarian* and *normic* concepts of diversity (Steel et al., 2018). To illustrate the difference, consider a situation in which racial diversity is the focus.^1^ The more racial categories that are represented in a group and the more evenly distributed the numbers of each, the more diverse the group is in an egalitarian sense. Diversity in an egalitarian sense therefore would be minimized when the group is composed entirely of individuals of the same racial category. In contrast, normic diversity identifies one category (white, for instance) as non-diverse, and judges the group to be diverse to the extent that it diverges from this non-diverse category. Egalitarian and normic concepts can disagree about the comparative diversity of groups. According to an egalitarian conception, a group composed solely of white people and another composed solely of black people both have zero diversity as there is no variation of the relevant attribute within either. However, according to a normic conception, the all black group is more diverse than the all white group because everyone in the all black group diverges from the non-diverse category while no one in the all white group does. Moreover, unlike egalitarian diversity concepts, normic diversity allows a single individual who differs from the non-diverse category to be labeled diverse, and expressions such as “diverse person,” “diverse student,” and so forth are often tell-tale signs that normic diversity concept is in play (Steel et al., 2018, p. 771). Thus, while egalitarian diversity refers to variety within a group, normic diversity points to a contrast between the group and a societal norm.

Two differences between egalitarian and normic diversity concepts are, we claim, especially important for understanding the diverse person’s burden. First, non-diversity in a normic sense entails homogeneity plus social privilege, as the non-diverse category is identified by its advantage and perceived normality in a larger reference population. In contrast, any homogeneous group is non-diverse in an egalitarian sense, whether it is composed of socially privileged individuals or not. As discussed below, social privilege is linked to psychological entitlement, which we argue plays an important role in generating the diverse person’s burden.

Second, the egalitarian concept of diversity neither asserts nor denies the existence of inequalities in the broader society.^2^ Consequently, one can conceive of diversity in an egalitarian sense, and hold beliefs about its positive effects, in a manner that utterly disregards, or even actively denies, the reality of social inequalities and discrimination along such lines as race, gender, and sexual orientation. To appreciate this point, imagine an ideally egalitarian world in which a variety of demographic groups exist but wherein no differences of socioeconomic, social and institutional status are present. In such a utopia, normic diversity could not exist, because there would be no privileged social category that could constitute the non-diverse norm. But egalitarian diversity would still be possible, since there could be groups containing people from a variety of demographic backgrounds, and this diversity might be beneficial as the business case for diversity suggests. A person who conceives of diversity in an egalitarian sense and is convinced of its value, then, might believe that society in general, or the specific organization they inhabit, approximates this utopian scenario.

The egalitarian concept of diversity, moreover, is closely linked to the business case for diversity, according to which demographic diversity increases the variety of task-relevant perspectives, ideas, or experiences, which in turn leads to superior results. The idea that demographic differences within groups can lead to epistemically valuable cognitive diversity has also been advocated by several philosophers (Anderson, 2006; Longino, 1990, 2002; Muldoon 2016) and is a central theme of Justice Lewis Powell’s influential opinion in the 1978 US Supreme Court case *Regents of the University of California v. Bakke* (Berrey, 2015). Cognitive diversity is thought to be beneficial by stimulating the innovative, critical thinking needed to succeed at complex, non-routine tasks. Hong and Page’s (2004) widely cited “Diversity Trumps Ability” theorem is often taken to support such claims. According to this theorem, a randomly selected (and thus more varied) group will almost always outperform a group of best performing individuals when the problem is difficult, all individuals are competent, the population of individuals from which group members are selected is cognitively diverse in task relevant ways, and the population and groups are sufficiently large (Page, 2007; cf. Hong & Page, 2004). Page has promoted practical applications of the “Diversity Trumps Ability” theorem in books aimed at popular audiences (Page, 2007, 2017), and his work has been influential within and without academia (Grim et al., 2019).

In the business case for diversity, both demographic and cognitive diversity refer to variety within the group, although with respect to differing sets of characteristics. Moreover, it is the interaction among differing cognitive resources within the group that is claimed to generate the epistemic benefit. Thus, such reasoning is naturally interpreted as relying on an egalitarian conception of diversity, in which diversity refers to within-group variation (cf. Steel et al., 2018, pp. 770–771). By contrast, normic concepts of diversity are typically associated with explanations, such as standpoint epistemology, according to which socially marginalized individuals are less prone to distorted reasoning and false beliefs on topics relating to oppression and discrimination (Harding, 2004, 2015; Intemann, 2010; Rolin, 2016; Wylie, 2011). Importantly, such explanations do not depend on variety within the group. For example, a group of black women may be better positioned to comprehend the intersecting effects of racism and sexism than a group of white men.

Consequently, pro-diversity beliefs couched in terms of the egalitarian diversity concepts can be consistent with obliviousness to or even denial of the existence of social injustices that may be related to the absence of diversity in a particular setting. Indeed, such beliefs may be conjoined with explanations of how social and economic inequalities are not unfair. For instance, the idea that homogeneity arises from innocent causes is suggested by the “Diversity Trumps Ability” theorem described above. This theorem applies to a setting in which homogeneous groups arise from a strict application of meritocracy (i.e., selecting only the individually best performing agents), while diverse groups are produced by randomly selecting from agents who pass a threshold of competency (Hong & Page, 2004). If taken as an argument for promoting diversity along socially salient lines like race or gender,^3^ this model suggests that the relevant question is: Why select members of an organization with an eye to diversity, rather than solely on the basis of merit? Such a framing suggests that meritocracy is the cause of homogeneity in the organization, rather than, say, pervasive stigmatization and exclusion of minorities, opportunity hoarding by socially advantaged groups, or socially homogeneous professional networks. Consequently, the “Diversity Trumps Ability” theorem is consistent with the assumption that absence of diversity is unrelated to broader social inequalities and injustices.^4^

That differs from a normic conception of diversity, which highlights the fact that when groups such as boards of directors, corporate executives, university departments, scientific teams, or members of parliaments are homogeneous they are often also privileged in terms of income, education, gender, and race. We suggest that privilege is relevant to the diverse person’s burden because of its tendency to foster psychological entitlement. Psychological entitlement is a theoretical construct that refers to a persistent sense of oneself as being more deserving of benefits than others. Importantly, psychological entitlement is not tied to a specific context in which one feels owed something, such as a pay raise for superior performance, but is a general sense of heightened deservingness across many domains. The most commonly used instrument for measuring psychological entitlement is the Psychological Entitlement Scale (PES) developed by Campbell and colleagues (2004). This scale consists of nine statements with which respondents can express agreement or disagreement on a 7-point scale (from strong agreement to strong disagreement). The statements include, “I honestly feel I’m more deserving than others,” “If I were on the Titanic, I would deserve to be on the *first* lifeboat,” and, “I feel more entitled to everything.”^5^ A number of studies using PES find that psychological entitlement predicts greater selfishness, rule breaking, and lack of empathy (Campbell et al., 2004; Yam et al., 2017; Zitek et al., 2010; Zitek & Schlund, 2020). For example, an online survey of respondents from the United States by Zitek & Schlund (2020) found positive associations between psychological entitlement and self-reported failure to follow public health guidelines relating to COVID-19.

For the present purposes, it is important to consider how psychological entitlement relates to privilege. A number of studies find that socioeconomic privilege tends to be associated with greater psychological entitlement (Piff, 2014; Côté et al. 2021). Given such findings, one would expect higher than average psychological entitlement among groups that are non-diverse in a normic sense. Other studies suggest perceived victimhood as a cause of psychological entitlement; for example, a study by Zitek et al., (2010) found that participants tended to score higher on PES after being asked to think about a situation in which they were wronged. Building on such findings, Grubbs and Exline (2016) propose a model in which psychological entitlement leads to exaggerated expectations of benefits that, when not satisfied, fuel perceptions of unfair treatment and victimhood, which in turn trigger an even stronger sense of entitlement, along with defensiveness and interpersonal conflict. A related study by Phillips and Lowery (2020) finds that privileged individuals tend to respond to evidence of inequities related to their own privilege by defensively describing ways in which they have suffered or been victims. In the context of Grubbs and Exline’s model, this result suggests that raising concerns about inequities linked to the absence of diversity in an organization whose members are socially privileged may reinforce psychological entitlement and provoke hostile reactions. Consider the implications of these ideas for pro-diversity beliefs and the diverse person’s burden.

We suggest that psychological entitlement is connected to the diverse person’s burden because of its propensity to foster beliefs that inequalities are not unfair. That is, beliefs that one’s privilege is to some significant extent unearned and linked to social injustices are obviously dissonant with the view of oneself as specially entitled to benefits. Thus, psychologically entitled people would be motivated to find explanations of why any inequalities that favor them are not unfair (cf. Zitek & Jordan, 2016). Such explanations highlight some distinguishing features of the advantaged individuals that, within the social context, are likely to be seen as justifying preferred treatment. In the context of diversity, one very likely explanation is that the inequality is due to merit. To illustrate this idea, imagine a group of senior executives at a major corporation composed almost exclusively of white heterosexual cis men. These men would likely be attracted to explanations which suggest that merit explains the homogeneity in their ranks. We frankly suspect that the widespread appeal of the “Diversity Trumps Ability” theorem as a model for understanding diversity is in large measure due to the fact that it carries just such a suggestion.

Such a mindset can lead to unfair expectations associated with the diverse person’s burden. The belief that selection by individual merit explains homogeneity while diversity is sought because of its potential to produce bonuses suggests that diverse people should go above and beyond to prove they belong. In short, psychological privilege provides a simple reason why members of groups that are non-diverse in a normic sense would be strongly inclined to hold beliefs that lead to the diverse person’s burden. Moreover, psychologically privileged people would be very resistant to explanations which suggest that their status is in any way unearned or tied to unfairness in society, and such suggestions might elicit hostile responses, as discussed in the previous section.

An analogy may be helpful for appreciating the above ideas. Anyone who has traveled by air has noticed the short lines for business class travelers and the long lines for others. The justification in this case would be that the business class travelers have paid a premium for greater convenience and comfort, an explanation that many people would accept as reasonable. Now consider someone who has not purchased a business class ticket, but who nevertheless wishes to go through the shorter business class line. Plainly, some additional justification will be expected for this. Perhaps the person has some special health condition that makes it difficult for them to stand, for instance. The business class denizens would likely regard it as absurd and obnoxious if the person produced no such special justification but demanded the right to proceed through the business class gate anyhow. And if a satisfactory justification were produced, then they would naturally expect the interloper to be grateful for the privilege that had been generously extended. This is the logic of the diverse person’s burden: if the unequal distribution is fair, then any equalization of that distribution, however partial, requires some justification. That produces an onus on the diverse person to demonstrate a justification for their presence not expected of non-diverse individuals, and furthermore generates expectations of gratitude.

There are several connections between the analysis of the diverse person’s burden given above and the literature reviewed in Sect. 2. Consider Anderson’s claim that the “diversity model” can promote racial stereotyping when detached from social justice concerns. Anderson’s “diversity model” is a version of the business case for diversity, which can encourage disregard of social inequities related to homogeneity as explained above in connection with Hong and Page’s “Diversity Trumps Ability” theorem. Our approach explains why beliefs that inequalities are fair are especially appealing to members of normically non-diverse groups, and why such beliefs can create expectations that diverse people should produce distinctive bonuses to justify their presence. Davis’ prejudicial credibility excesses are a likely way that diversity bonuses might be expected to arise, that is, from special abilities or knowledge stereotypically associated with the diverse person’s demographic group. Examples of the oppressed explaining their own oppression to the oppressors, discussed by Davis and Berenstain, are one type of situation in which such expectations can occur, but hardly the only one. For example, think of an Asian American person hired into a New York advertising firm that wishes to do more business in China.

According to the proposal advanced here, members of normically non-diverse groups tend to have enhanced levels of psychological privilege, which motivate beliefs that inequalities that advantage them are fair, and which in turn prompt expectations that diverse individuals produce special bonuses to justify their inclusion. This dynamic can unfold in business, academia, and many other contexts. Moreover, informing privileged individuals about inequalities in their own social environment might, surprisingly enough, tend to strengthen their beliefs that those inequalities are fair.

A study by Côté et al., (2015) suggests that reduced generosity among economically advantaged individuals arises only in the context of high perceived inequality. Côté et al. offer three main lines of argument for this claim. First, they note that it is supported by previous research, which has found a negative correlation between wealth and generosity in highly economically unequal locations, such as California, but not in less economically unequal ones, like Germany, Japan, and the Netherlands (Côté et al., 2015, 15,841). Second, Côté et al. analyzed a nationally representative data set of individuals living in the United States (*n* = 1,498) that was produced by the Measuring Morality survey.^6^ Respondents reported household income along with other demographic information and then took part in a dictator game, in which respondents were randomly selected to decide how many of 10 raffle tickets to share with another survey respondent (Côté et al., 2015, p. 15,839). Consistent with previous research, generosity in the dictator game was negatively correlated with income only in states with relatively high levels of economic inequality. Finally, Côté et al. conducted an online experiment, in which participants from the United States were randomly assigned either to a group whose members were informed that their state is highly economically unequal or to a group that was presented with information suggesting their state was relatively economically equal. As in the survey, household income along with other demographic information was recorded, and the participants played a dictator game to measure generosity. In this experiment, participants who were informed that they lived in a highly unequal state were less generous than those who were told that their state was relatively equal. Côté et al. suggest two possible explanations for their findings (Côté et al., 2015, p. 15,841).^7^ The first is that a perception of being advantaged in a highly unequal society prompts people to explain these inequalities by emphasizing their own merit in comparison to those who are less well off. A belief in such explanations could then promote psychological entitlement and selfishness. The second explanation is that a perception of the dire straits of the less well off in a highly unequal society could prompt concern about one’s own risk of falling into the wrong side of a starkly unequal social divide, and thereby lead to greater selfishness. The first of these two explanations seems to fit better with Côté et al.’s data, however, because perceived risk of destitution would presumably also motivate selfishness among lower income people. Yet Côté et al., (2015, p. 15,841) report that being informed that one’s state is highly unequal only decreased generosity among higher, not lower, income participants.

In sum, Côté et al.’s work suggests that educating privileged individuals about inequalities might have the unintended consequence of increasing their sense of entitlement and decreasing their inclination to be generous. That coincides with the discussion of active ignorance from the previous section. Spurious explanations of how inequalities are fair may arise as an active response to awareness of inequalities, rather than from simply being uninformed. And these are precisely the sorts of beliefs that, according to our proposal, drive the diverse person’s burden.

## Where to go from here?

In this section, we consider what might be done about the diverse person’s burden in light of our proposed analysis of it. We explore the implications of our proposal on this issue from structural, institutional, and individual lenses, in that order. By structural, we refer to the extent of historical and current inequalities in wealth and access to opportunities within a society and the social, economic, legal, and political causes of those inequalities. By institutional, we refer to the internal structure and habitual workings of particular organizations, such as corporations, universities, hospitals, or government agencies. At the individual level, we are concerned with individual persons operating within broader social-structural and institutional contexts.

### Structural changes

Our proposal suggests that greater social and material inequality within a society accentuates the diverse person’s burden. As noted in the previous section, socioeconomic advantage tends to be associated with heightened psychological entitlement (Piff, 2014; Côté et al. 2021). Moreover, those who were born into wealth tend to exhibit more psychological entitlement than those who became wealthy in their own lifetimes (Côté et al., 2021). This suggests that more extreme and entrenched social inequalities can lead to a stronger sense of psychological entitlement among the advantaged, and consequently to a stronger motivation to seek out explanations of how inequalities that benefit them are fair. According to our analysis of the diverse person’s burden, marginalized individuals in privileged social spaces would face more intense expectations to produce distinctive bonuses in more starkly unequal societies.

As a result, our argument suggests that reducing social inequalities in a society would also have the long run effect of blunting the diverse person’s burden. Structural measures that promote social equality include such things as progressive income tax, taxes on inherited wealth, prosecution of tax evasion among the wealthy, universal access to quality healthcare and education, and rigorous prevention of racial and other forms of discrimination in the legal system, housing, and employment (cf. Piketty, 2020). There are, of course, many reasons to favor equality-promoting measures such as these besides concerns about the diverse person’s burden. From a structural perspective, then, the diverse person’s burden is one social injustice among many that is likely to rear its ugly head in highly unequal societies. However, one can also consider structural measures more specifically focused on reducing the diverse person’s burden.

Legal precedents can play an important role in maintaining unequal social structures. As noted above, the *Bakke* decision created a legal context in the United States wherein remedying past or ongoing discrimination could not be a rationale for measures to increase the representation of marginalized people in education, employment, or elsewhere. The *Bakke* decision is relevant to the diverse person’s burden because it is related to explanations of social inequalities, a topic that links up with ideology. The current philosophical literature on ideology distinguishes between cognitivist and more practice-oriented approaches. Whereas the first claims that ideologies are constituted by a widely shared set of beliefs (Shelby, 2003), the latter puts more emphasis on ideological practices (Haslanger, 2017; Táíwò, 2018). According to Shelby (2003, pp. 183–184), ideology is a system of beliefs that: (i) contains epistemic distortions (i.e., false or misleading claims), (ii) functions to reinforce social inequalities, and (iii) is widely accepted among certain socially advantaged groups due to motivated reasoning. According to practice-oriented accounts, racism, hetero-sexism, classism, ableism and similar ideologies are not merely sets of beliefs, but also consist of social practices and conventions embedded in an unequal social and economic system (Haslanger, 2017, p. 13). To see the contrast between cognitive and practice-oriented accounts, consider Táíwò’s (2018) point that an ideology may exert power not because it is widely believed but due to incentives (e.g., fear of ostracism or reprisal) that compel people to publicly support it.

Consider the cognitivist and practice-oriented accounts of ideology in relation to the precedent set by the *Bakke* decision. *Bakke*’s prohibition on redress of discrimination as a reason for increased diversity in university admissions encourages beliefs that racism and discrimation are matters of the past. Such beliefs arguably satisfy Shelby’s three criteria of ideology: they are distorting insofar as they encourage ignorance of discrimination, they have the effect of buttressing an unequal status quo, and motivated reasoning plausibly explains the prevalence of such beliefs among socially advantaged people. Táíwò’s suggestion that ideology may prompt people to express agreement with claims they privately reject is also relevant here. Given the *Bakke* decision, anyone mounting a legal defence of diversity initiatives at a university in the United States would be constrained to do so in terms of the business case for diversity, regardless of their private views of the matter. Moreover, the practice of treating the business case as the primary argument for diversity has extended beyond the borders of the United States to countries where the *Bakke* decision carries no legal force. All this suggests the *Bakke* decision as a target for structural change that would be relevant to the diverse person’s burden.

### Institutional remedies

Whereas structural changes impact people across many different social settings and locations, institutional measures focus on the rules, practices, and norms within a specific organization. Since the diverse person’s burden may vary depending on the institution and the marginalized people in question, we consider a specific case. A review by Chaudoir et al., (2017) examines research on interventions to reduce the stress among “sexual minorities”, that is people who are part of the LGBTQIA + community, implemented in a variety of settings, including schools, universities, and workplaces. They distinguish between interventions that aim to reduce causes of stress and measures that promote coping strategies to mitigate these stresses (Chaudoir et al., 2017, p. 589).^8^ Interventions in the former category tend to be directed at heterosexual cis individuals (e.g., anti-bullying policies, or LGBTQIA + educational training), while queer people would be the primary foci of interventions of the latter sort (e.g., mindfulness training, support groups). These interventions can be considered “institutional” in our usage of this term insofar as they could become embedded in the regular practice of an organization (e.g., a workplace might routinely mandate LGBTQIA + educational training, or create a LGBTQIA + support group).

Our diagnosis of the diverse person’s burden naturally suggests including cis heterosexual privilege as topic for training interventions targeted at cis heterosexual individuals, and this was indeed a focus for some of the interventions reviewed by Chaudoir et al., (2017, p. 605; cf. Case and Stewart 2010). An organization that offered such training on a regular basis would, in effect, be making an institutional commitment to promoting what, in Sect. 4.3, we call positional awareness. As explained in further detail below, positional awareness refers to an understanding of ways in which one’s position in society carries unearned advantages. Although we discuss positional awareness as an individual virtue, it is important to recognize that institutions have a certain amount of power in promoting virtues among its members and in society more broadly.

However, there are limitations to what diversity training can be expected to accomplish. People may be resistant to such training for reasons discussed above, and even when diversity training succeeds in changing people’s outlooks, this might not translate into action. Consequently, additional institutional measures beyond diversity training are often needed. One possibility here consists of providing support for sub-organizations dedicated to the needs of specific marginalized groups. Such advocacy groups can provide support for marginalized individuals in coping with the diverse person’s burden. In addition, if adequately integrated into the organization, they may also help to reduce the prevalence of stressors by drawing attention to problems or, more proactively, advancing ideas about how the organization could be improved. To play these roles, advocacy groups need to be properly supported, not marginalized, and able to regularly participate in decision-making venues. There are a number of pitfalls for such efforts that can arise in practice. For instance, Táíwò (2020) cautions that a naïve imperative of “making space” for the marginalized and “stepping aside” can conceal dominant social structures, harm marginalized groups, and diminish the privileged group’s responsibility. Developing practices that productively integrate advocacy groups into an organization, then, can be an important aspect of institutional reform.

### Individual Virtue

Given our analysis of the diverse person’s burden, the key question from the perspective of individual virtue is how to avoid harmful effects of psychological entitlement among privileged individuals. This is primarily a responsibility for people who are privileged along some important dimension, such as race, gender, or wealth. We suggest a virtue we call *positional awareness*. This involves being aware of ways in which one’s social position within a broader social system is privileged, and being aware of how this privilege may foster psychological entitlement and lead to ignorance and bias. Practicing this virtue entails taking time to learn about social inequalities in which one finds oneself on the advantaged side, considering how one’s social position may motivate beliefs of narratives that would justify these inequalities, and taking steps to mitigate the behavioral effects these beliefs may have.^9^

For example, consider a high-income white male living in a settler colonial state such as Australia, Canada, or the United States. For such a person, practicing the virtue of positional awareness would involve making efforts to learn about historical and ongoing systemic racial, gender, and economic inequalities that likely contributed to his privileged position within society. His access to opportunities was likely tied to his parents’ wealth, which in turn was enhanced by a history of social and economic policies that systematically advantaged whites, often at the expense of Indigenous people, black people, and others. Likewise, the advantage of being viewed as the social norm in high status educational and professional spaces was likely a contributor to his success. Moreover, this position may result in obliviousness to experiences of those who are not similarly perceived as the norm. And the emphasis in Western culture on linking a person’s value to demonstrated merit within their chosen profession provides ample motivation for beliefs that reinforce such ignorance and suggest that he has earned everything through talent and hard work.^10^

Positional awareness, then, combats what Dotson refers to as situated ignorance (Dotson, 2012a, pp. 31–32), that is, ignorance resulting from a social position that makes it easy to disregard information and ideas that draw attention to social injustices. This ease of ignorance may be due to the fact that the information and ideas in question are marginalized in mainstream discourse, or deliberately covered up, as in the case of the 1921 Tulsa race massacre (Ellsworth, 2021). But according to our account of the diverse person’s burden, it is also related to enhanced psychological entitlement among privileged people. Those who see themselves as especially deserving of benefits are likely to find an honest look at ways in which they reside on the advantaged side of social inequalities highly uncomfortable. Such cognitive dissonance can motivate evasive mental maneuvering such as seeking out reasons why those inequalities are inevitable, or not really that bad, or why it is offensive to even raise the topic, or why *other*––supposedly more righteous!––people are really the privileged, ignorant and biased villains. Distorting narratives of these kinds can prevent people from drawing obvious conclusions from available information. Ellsworth’s (2021) account of the 1921 Tulsa race massacre depicts multiple examples of these processes among white Tulsans. For example, an inquiry in the aftermath of the massacre held the black community responsible, while some white members of a commission created to address the topic in the late 1990s pushed conspiracy theories pinning the blame on non-Tulsans. Overcoming situated ignorance, then, can be very difficult and, if successful, is likely to be a process that plays out over an extended period of time.^11^

The purpose of positional awareness is not to encourage feelings of guilt. After all, a person’s unearned advantages are typically tied to a vast history and social system far beyond the control of any individual. And it is normal for people to pursue opportunities that society and life offers them. Instead, the point is to recognize the strong potential for systemic advantages to foster psychological entitlement and skew beliefs towards spurious justifications of the status quo, and therefore to make a conscious effort to correct for this. Knowledge that one may be biased in this manner should cause one to be skeptical of one’s own beliefs in narratives that seek to justify social inequalities, especially if one’s social and professional circles are primarily composed of privileged people similar to oneself. One should also be alert to the potential for insidious behavioral effects of these biases, for instance, in the form of expectations that marginalized co-workers deliver “bonuses” stereotypically associated with their social categories. Active steps, then, would be in order to counter such tendencies.

These active steps should not be limited to self-reflection. As discussed in 4.1. and 4.2, social structures and institutions play an important role in the diverse person’s burden. These structures and institutions are influenced by individuals who can support institutional and structural changes. As a privileged person, one should make efforts within one’s institution to address the diverse person’s burden. This includes supporting and highlighting often invisible diversity work (as Berenstain has defined it), advocating for proper recognition of these works as well as taking on a fair share of service work.

We suggest two caveats regarding the virtue of positional awareness. The first advises against moral smugness. It is easy to find examples of absurd narratives promoted to justify social inequalities, such as characterizations of European colonialism “as a civilizing mission” (Piketty, 2020, p. 270). And it can be very tempting to take such cases as evidence of one’s own moral superiority. However, a more reasonable inference is that such cases illustrate the powerful appeal of self-serving ideologies designed to justify inequalities––and this should give you pause. If other humans often engage in such chicanery, then you too may be prone to similar foibles. By analogy, suppose the vast majority of people commit an error on a cognitive test. This information should make you think that you would be likely to make similar errors. So, patent failings of others to practice the virtue of positional awareness should be an occasion for self-reflection, not arrogance or complacency.

The second caveat is to avoid seeking self-exemptions. Not every privileged person is an embodiment of the “mythical norm” of financially secure, cis-gendered, heterosexual, able bodied, white male. People obviously can be socially privileged in some aspects of their social position but not in others, for example, a low-income white man, a homosexual white woman, or a wealthy black man. And practically everyone has suffered tribulations in one form or another. However, a key aspect of practicing the virtue of positional awareness is not treating one’s hardships as a get out of jail free card. For example, a gay white man may have suffered discrimination relating to his sexual orientation, while still having enjoyed social privileges associated with being a white male. As such, he can practice the virtue of positional awareness. Of course, some are privileged more systematically and along more dimensions than others, and the virtue of positional awareness is, consequently, especially important for those fortunate sons. However, just as almost everyone has suffered misfortunes, most have benefited from some events and aspects of society over which they had little control. The virtue of positional awareness entails a focus on the latter rather than the former.

The virtue of positional awareness can be contrasted with Fricker’s (2007, 2010) proposed virtue of testimonial justice. The aim of Fricker’s virtue is to avoid testimonial injustice, wherein a hearer unfairly discounts a speaker’s testimony due to prejudice against the speaker’s social identity. Fricker proposes that people should consider how aspects of their identity, such as their gender or race, that might predispose them to unfairly discount the testimony of others (Fricker, 2007, p. 91), should be alert to contextual cues that they may be about to commit testimonial injustice (Fricker, 2010), and should make efforts to counteract such prejudices by revising credibility judgments upward (2007, pp. 91–92). Alcoff (2010, p. 132) and Anderson (2012, p. 168) object to Fricker’s proposal on the grounds that prejudices and biases often operate below the level of one’s awareness, so people are likely to be unaware that they are committing testimonial injustice and would be unsure of how far to revise their credibility judgments even if they were. Such considerations are often taken to indicate that a focus on individual virtues is insufficient for addressing epistemic injustices, and that structural and institutional measures are also necessary (Alcoff, 2010; Anderson, 2012; cf. Fricker, 2010, pp. 165–166).

As should be apparent from Sect. 4.1 and 4.2, we have no objections to structural and institutional approaches to the diverse person’s burden. Moreover, our proposed virtue of positional awareness differs from Fricker’s virtue of testimonial justice in ways that are relevant to the criticisms raised by Alcoff and Anderson. Positional awareness starts from considering salient aspects of one’s social position, such as race, gender, class, and sexual orientation, which are typically much more readily observed than one’s prejudices. The recommended measures are to become more educated about the history and current social inequalities that relate to ways in which one’s own social position is privileged, and to consider ways in which this might motivate one to believe rationalizations of inequalities. These are volitional acts that can be deliberately undertaken and that are intended to reduce psychological entitlement among privileged people. Given our proposal, that reduction would be expected to alleviate the diverse person’s burden. While Fricker does draw attention to the fact that one’s identity might predispose one to unfairly discount a speaker’s credibility, her virtue of testimonial justice primarily focuses on catching oneself in the act of being biased and making adjustments in the moment. In contrast, positional awareness emphasizes taking actions in advance that make one less susceptible to self-serving narratives and stereotypes that lead to the diverse person’s burden.

## Conclusions

Dotson (2012) points out that efforts to avoid one type of injustice sometimes inadvertently inflict another. We have advanced the analogous claim that pro-diversity beliefs can sometimes have the negative effect of generating unfair expectations for marginalized people, a phenomenon that we refer to as the “diverse person’s burden”. We suggest that the concept of normic diversity is helpful for understanding the diverse person’s burden because it links non-diversity to socioeconomic privilege, which research suggests is associated with psychological entitlement. We suggest that psychological entitlement motivates privileged individuals to believe that inequalities that benefit them are fair, and that such beliefs can in turn can lead to the diverse person’s burden. For example, the belief that racial homogeneity in an organization is due to meritocracy prompts expectations that racially marginalized individuals should demonstrate some distinctive bonus to justify their inclusion. We consider structural and institutional measures that may be helpful for addressing the diverse person’s burden, and we propose an individual virtue of positional awareness that involves making efforts to understand ways in which one is advantaged.

## Data Availability

Not applicable.

## Footnotes

1 Of course, multiple attributes (e.g., race, gender, religion, etc.) are relevant in most real-life circumstances.

2 The label “egalitarian” only refers to the equal distribution of categories within the group, not to equality in any broader, societal sense.

3 Hong and Page’s theorem pertains to cognitive diversity (specifically, diversity in perspectives and search heuristics), and hence it is unclear to what extent it is relevant to diversity at the level of race, gender, social class, and so forth (cf. Hong & Page, 2004, pp. 16,385–16,386; Page 2017, p. 169). Consequently, it is debatable whether Hong and Page’s theorem should be viewed as an argument for diversity in the latter, more social sense of the term.

4 Anderson (2013, pp. 72–73) makes similar observations regarding Schelling’s famous (1978) model of how slight preferences for neighborhoods in which one’s own group is in the majority can lead to housing segregation.

5 An anonymous reviewer suggests that, because statements like, “I feel more entitled to everything,” seem so obviously asocial, genuinely psychologically entitled people might express strong disagreement with them so as not to appear selfish. This amounts to a concern that PES is *insensitive*, that is, that it tends to underestimate how psychologically entitled people actually are. While this is a legitimate concern, the fact that PES correlates with variables such as selfishness and rule breaking indicates that it is not wholly insensitive. And if PES is insensitive to some degree, then the research results discussed here would tend to *underestimate* the effects of psychological entitlement.

6 For more about this survey, please see: https://kenan.ethics.duke.edu/attitudes/resources/measuring-morality/.

7 Côté et al. divide the first explanation into two: one in which advantaged people attribute negative characteristics to those who are less well off, and another in which they believe that inequalities are due to meritocracy. However, these explanations strike us as substantially the same, because taking oneself to be more meritorious than others entails a negative assessment of others in comparison to the self (e.g., others are less hard working or talented) and vice versa.

8 Chaudoir et al. define “sexual minorities” to refer only to lesbians, gay men, and bisexual people. However, our discussion is generalized to all individuals who are part of the LGBTQIA + community.

9 The virtue of positional awareness can be seen as the opposite of an epistemic vice (Kidd et al., 2021). For instance, Tanesini (2021) discusses arrogance as an epistemic vice for privileged groups. She defines arrogance as a defensive form of self-esteem, highlights the low threshold for evidence privileged groups will employ for self-serving narratives, and how easy it is to maintain these narratives through societal and institutional selection processes.

10 Note that the epistemic failings of this case might also be described in Medina’s (2013) account of active ignorance, which emphasizes the vices of closed-mindedness, the active avoidance of other perspectives and related evidence, and epistemic arrogance, the propensity for thinking of oneself as an epistemic authority. Both of these vices are rooted in structural oppression and help to maintain certain types of oppression. Importantly, on Medina’s account, people who are actively ignorant are morally and epistemically blameworthy for lacking certain knowledge and for maintaining systematic “bodies of ignorance” (2013, pp. 39).

11 An anonymous referee asks how people with an enhanced sense of psychological entitlement could be motivated to develop the virtue of positional awareness, given that doing so would likely be uncomfortable for them. We think the most likely motivation is a sincere commitment to social justice, anti-discrimination and the like. For one who is committed to social justice and anti-discrimination, positional awareness is obviously a desirable virtue to cultivate, even if doing so is difficult or uncomfortable.
